# The "Begin Exploring Fertility Options, Risks and Expectations" (BEFORE) decision aid: development and alpha testing of a fertility tool for premenopausal breast cancer patients

**DOI:** 10.1186/s12911-019-0912-y

**Published:** 2019-10-28

**Authors:** Brittany Speller, Kelly Metcalfe, Erin D. Kennedy, Marcia Facey, Ellen Greenblatt, Adena S. Scheer, Ellen Warner, Anil Abraham Joy, Frances C. Wright, Nancy N. Baxter

**Affiliations:** 1 Department of Surgery, Li Ka Shing Knowledge Institute, St. Michael’s Hospital, 040-16 Cardinal Carter Wing, 30 Bond Street, Toronto, ON M5B 1W8 Canada; 2 Lawrence S. Bloomberg Faculty of Nursing, University of Toronto, Toronto, Canada; 3 Women’s College Research Institute, Women’s College Hospital, Toronto, Canada; 4 Institute of Health Policy, Management, and Evaluation, Dalla Lana School of Public Health, University of Toronto, Toronto, Canada; 5 Department of Surgery, Mount Sinai Health System, Toronto, Canada; 6 Leslie Dan Faculty of Pharmacy, University of Toronto, Toronto, Canada; 7 Mount Sinai Fertility, Department of Obstetrics and Gynecology, Sinai Health System, Toronto, Ontario Canada; 8 Department of Medical Oncology, Odette Cancer Center, Sunnybrook Health Sciences Centre, Toronto, Ontario Canada; 9 Department of Oncology, University of Alberta, Cross Cancer Institute, Edmonton, Alberta Canada; 10 Department of Surgery, Sunnybrook Health Sciences Centre, Toronto, Ontario Canada

**Keywords:** Decision aid, Oncofertility, Fertility preservation, Breast cancer, Decision-making, Alpha testing

## Abstract

**Background:**

Premenopausal breast cancer patients are at risk of treatment-related infertility. Many patients do not receive sufficient fertility information before treatment. As such, our team developed and alpha tested the Begin Exploring Fertility Options, Risks, and Expectations decision aid (BEFORE DA).

**Methods:**

The BEFORE DA development process was guided by the International Patient Decision Aids Standards and the Ottawa Decision Support Framework. Our team used integrated knowledge translation by collaborating with multiple stakeholders throughout the development process including breast cancer survivors, multi-disciplinary health care providers (HCPs), advocates, and cancer organization representatives. Based on previously conducted literature reviews and a needs assessment by our team – we developed a paper prototype. The paper prototype was finalized at an engagement meeting with stakeholders and created into a graphically designed paper and mirrored online decision aid. Alpha testing was conducted with new and previously engaged stakeholders through a questionnaire, telephone interviews, or focus group. Iterative reviews followed each step in the development process to ensure a wide range of stakeholder input.

**Results:**

Our team developed an 18-page paper prototype containing information deemed valuable by stakeholders for fertility decision-making. The engagement meeting brought together 28 stakeholders to finalize the prototype. Alpha testing of the paper and online BEFORE DA occurred with 17 participants. Participants found the BEFORE DA usable, acceptable, and most provided enthusiastic support for its use with premenopausal breast cancer patients facing a fertility decision. Participants also identified areas for improvement including clarifying content/messages and modifying the design/photos. The final BEFORE DA is a 32-page paper and mirrored online decision aid (https://fertilityaid.rethinkbreastcancer.com). The BEFORE DA includes information on fertility, fertility options before/after treatment, values clarification, question list, next steps, glossary and reference list, and tailored information on the cost of fertility preservation and additional resources by geographic location.

**Conclusion:**

The BEFORE DA, designed in collaboration with stakeholders, is a new tool for premenopausal breast cancer patients and HCPs to assist with fertility discussions and decision-making. The BEFORE DA helps to fill the information gap as it is a tool that HCPs can refer patients to for supplementary information surrounding fertility.

## Introduction

Future fertility is a common concern for many premenopausal breast cancer patients at risk of treatment-related infertility [1]. Systemic therapy, while a potentially lifesaving treatment, can be detrimental to breast cancer patients’ fertility [2]. In addition, some patients require hormone therapy for five and up to 10 years after all other treatments are completed [3], during which time their natural fertility declines. For women at risk of infertility after breast cancer treatment, the decision to complete fertility preservation (FP) before treatment is a time and preference-sensitive decision that requires consideration of potential treatment delays, cost of FP, future childbearing plans, and uncertainty surrounding survival, all during an emotional time following a cancer diagnosis [4, 5]. Additionally, many patients are not provided adequate, targeted fertility information or referrals to reproductive specialists prior to decision-making even though they have fertility concerns [5, 6].

Decision aids (DAs) are evidence-based tools that help prepare users for preference-sensitive decision-making by providing information on a specific health condition. Decision aids highlight the benefits and risks, probabilities, and uncertainties of the different options for the health condition allowing patients the opportunity to clarify their values and consider each option based on their own preferences before making an informed decision [7]. In a 2017 review of 105 DA studies, Stacey et al. [8] found that DAs also have an impact on patient outcomes and decisions. Specifically, patients who used DAs were more likely to choose conservative treatment options over invasive options in elective surgeries and had decreased decisional conflict due to feeling less uninformed. The highest-quality evidence included in the review revealed that patients who use DAs have increased knowledge, consistency between informed values and the option chosen, and accuracy of risk perception [8]. Due to the high-level of evidence highlighting the benefits of DAs during decision-making, multiple recommendations exist regarding the use and development of oncofertility decision support resources to help disseminate fertility information and assist patients in making informed decisions in partnership with their health care providers (HCPs) [5, 9–12].

Oncofertility DAs are available for premenopausal breast cancer patients in Australia [13], the Netherlands [14], and Germany [15]. Additional, oncofertility DAs are being evaluated for young women with cancer in United States [16] and United Kingdom [17]. At the time of DA development, the publicly available DAs included a printable PDF (paper) in Australia [13] and an interactive website in the Netherlands [14]. No publicly accessible DAs are available in both paper and online formats in both English and French languages. Additionally, existing DAs that are publicly accessible contain information that is country specific, contain varying depths of information, and do not separate out the fertility option success rates by the patients’ age or depict the pregnancy success rates by age using visual graphic displays. As such, we aimed to create and alpha test the Begin Exploring Fertility Options, Risks, and Expectations (BEFORE) DA for premenopausal breast cancer patients in Canada.

## Methods

This study received Research Ethics Board approval from St. Michael’s Hospital (REB #15–220).

### Guiding frameworks

The BEFORE DA was developed following a modified systematic development process outlined by the International Patient Decision Aid Standards (IPDAS) (i.e., our team collaborated with a range of stakeholders instead of forming a steering group) [18] and was guided by the Ottawa Decision Support Framework (ODSF) and Ottawa Decision Support Tutorial [19].

The IPDAS Collaboration was created in 2003 and is comprised of international researchers, HCPs, and other stakeholders. The collaboration aims “*to enhance the quality and effectiveness of patient decision aids by establishing a shared evidence-informed framework for improving their content, development, implementation, and evaluation*” [20]. Using a Delphi consensus process, the collaboration established internationally approved DA criteria spanning across 12 quality dimensions [21]. Among the multiple phases of work completed by the IPDAS, including the creation of an IPDAS instrument to assess the quality of DAs and developing minimum standards for certifying DAs, the group conducted systematic reviews outlining the evidence for the 12 quality dimensions [20]. The reviews were published in 2005 and updated in 2013 to highlight the theoretical foundation of each criteria and recommendation. The reviews focused on the development process for DAs, disclosing conflicts of interest, the information in DAs, the use of scientific evidence, the balanced presentation of information and probabilities, values clarification, using personal stories, health literacy, shared decision-making using DAs, DAs on the Internet, and the dissemination and implementation of DAs [20]. Our team used the updated reviews and IPDAS criteria to guide the development of the BEFORE DA.

In 1995, the Patient Decision Aids Research Group from the Ottawa Hospital Research Institute created the ODSF, one of the first frameworks to assist with the development of DAs [19]. The ODSF is based on theories and concepts from general and social psychology, social support, decision analysis, decision conflict, values, and self-efficacy. Overall, the ODSF states that decisional needs among patients will have an impact on their decision quality, which will then impact their behaviour, actions, emotions, health outcomes, and use of health services. Decision support, such as DAs and counselling, can improve the decision quality among patients by addressing their unresolved decisional needs [22]. The Ottawa Decision Support Tutorial was created in 2007 and updated in 2015 and is based on the ODSF. The tutorial provides information and self-assessments on decision support to help educate HCPs [19]. Members of our team reviewed the tutorial prior to the BEFORE DA development to understand decision support in the context of premenopausal breast cancer patients making fertility-related decisions prior to cancer treatment.

The team chose the IPDAS and ODSF to guide development of the BEFORE DA as they are commonly used in the development of DAs for preference-sensitive decisions [18] and to ensure compliance with internationally recognized standards for the development of high quality DAs. The frameworks advocate for the inclusion of a range of stakeholders throughout the DA development process and as such our team used an integrated knowledge translation (iKT) approach [23] during development. Our team collaborated with breast cancer patients who had made fertility-decisions prior to treatment, multi-disciplinary HCPs who provide care to premenopausal breast cancer patients (i.e., reproductive specialists, surgical oncologists, medical oncologists, social workers, nurses, general practitioners), a patient educational specialist, DA and cancer survivorship experts, advocacy group representatives from Rethink Breast Cancer, Young Adults Cancer Canada, and Cancer Knowledge Network, who provide support and information to cancer patients, and cancer organization representatives from Cancer Care Ontario and the Canadian Cancer Society, who provide information to cancer patients and guidance to HCPs.

### Development process

The goals of the BEFORE DA were established by the research team and verified with breast cancer survivors and multi-disciplinary HCPs: (1) to provide information to premenopausal breast cancer patients on the available fertility options prior to treatment that may cause infertility; (2) to assist premenopausal breast cancer patients in making fertility-related decisions that align with their values; and (3) to act as an adjunct to HCP consultation and to help premenopausal breast cancer patients and oncology HCPs discuss treatment-related fertility risks and make FP decisions.

Following the guiding frameworks, the team completed two systematic literature reviews: (1) the barriers and facilitators to FP discussions and decision-making [24] and (2) existing oncofertility decision support resources [25]. After the literature reviews, three sets of needs assessment interviews were conducted with: (1) premenopausal women previously diagnosed with cancer to explore their experience with fertility decision-making; (2) HCPs to understand the barriers faced when discussing fertility [26]; and (3) premenopausal breast cancer survivors and HCPs to evaluate existing oncofertility DAs (including the Australian [13] and Dutch DA [14]) and educational materials to determine key information needed during FP decision-making [27].

**Fig. 1:**
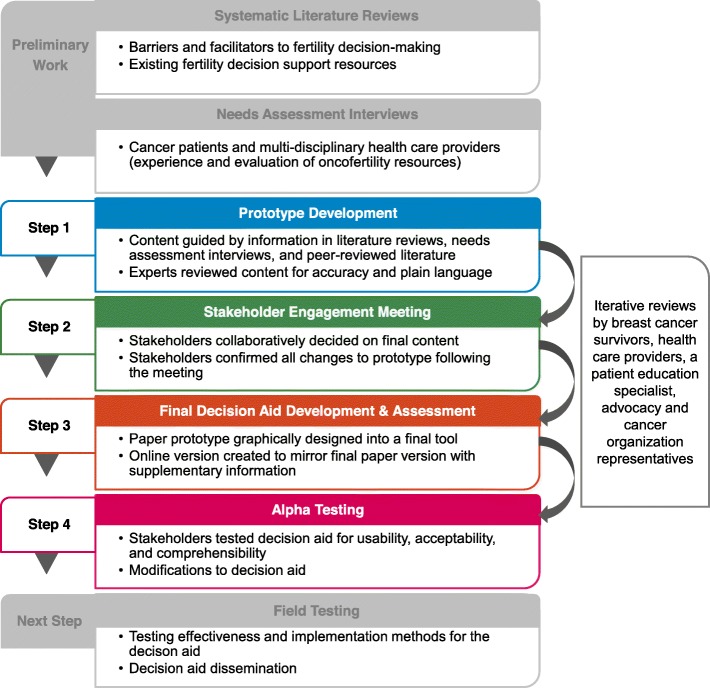
BEFORE (Begin Exploring Fertility Options, Risks, and Expectations) decision aid development process

The following paragraphs outline the development process in more detail including the paper prototype development, a stakeholder engagement meeting to create the final paper and online BEFORE DA, and alpha testing of the paper and online BEFORE DA. During the BEFORE DA development process, oncology and fertility content experts, breast cancer survivors, multi-disciplinary HCPs, and a patient education specialist also provided four iterative reviews of the paper and online DA and shared their general insights on ways to improve the DA (e.g., wording and design modifications) (Fig. 1).

### Step 1 –prototype development

Our team used the results from the systematic literature reviews and the needs assessment interviews to guide the development of the BEFORE DA paper prototype. The sections for inclusion in the prototype were based on aspects of the Australian and Dutch DAs [13, 14] and other oncofertility educational materials that all or the majority of breast cancer survivors and multi-disciplinary HCPs perceived as useful [27]. For two additional sections in the prototype (explicit values clarification method to help patients weigh the pros and cons of their decision and presentation of the fertility options success rates and risks), our team developed them in various formats to capture further feedback from a range of stakeholders on the preferred format. Specifically, the explicit values clarification method, designed to assist patients in identifying the FP option they prefer by allowing them to contemplate the desirability and attributes of each option [28], was created in four different formats (e.g., pros and cons list with pre-listed values, blank pros and cons list, sliding scale, and Likert-style questions) based on adaptations from existing DAs [13, 14, 29, 30] (Additional file 1). The pregnancy success rate statistics for each fertility option were created as pictographs in line with the Ottawa Decision Support Tutorial [19] and graphically as another option outlined by the IPDAS [31].

Our team reviewed the literature and consulted with content experts in oncology and fertility to identify the most up-to-date and applicable literature on fertility, pregnancy post-treatment, the health of children born to cancer survivors and using preserved embryos and eggs [32–36], the risk of infertility from cancer treatments [37, 38], pregnancy success rates and risks for the fertility options [36, 37, 39–42], and psychosocial aspects of fertility in survivorship [43, 44]. In addition, information was gathered from organizations that provide credible information to patients (Canadian Cancer Society [45], Fertile Future [46], American Society for Reproductive Medicine [47], Breastcancer.org [48]) as well as the American Society of Clinical Oncology (ASCO) fertility guidelines from 2006 and 2013 [49, 50]. The ASCO guidelines were updated again in 2018 [2], however the update did not prompt any significant changes to the existing guidelines. When inconsistences, ambiguities, and gaps in the literature existed, we sought the consensus of content experts in the fields of fertility and oncology.

During development of the BEFORE DA our team of experts in the fields of breast cancer and decision-making held multiple meetings to determine the format and design of the DA. Following development of the DA prototype, the team identified expert content reviewers through purposeful sampling. Initial reviewers included a reproductive specialist, a surgical oncologist, two medical oncologists, and a patient education specialist. Each reviewer electronically received the DA prototype and reviewed the medical content to ensure clinical accuracy and the use of plain language. Reviewer feedback resulted in content and design modifications to the prototype.

### Step 2 – stakeholder engagement meeting

Following prototype development, the team held a one-day stakeholder meeting to share results of the multi-prong oncofertility study, discuss the best way to present information in the DA, gather general feedback on the DA, and generate recommendations for strategic dissemination and continual updating of the DA. Invited stakeholders included breast cancer survivors, multi-disciplinary HCPs, a patient education specialist, DA experts, advocates, and cancer organization representatives. Purposive sampling was used to identify meeting participants who represented a range of perspectives from various professions, organizations, and geographic locations. The team also utilized snowball sampling [51] by asking invited participants who were unable to attend the meeting to recommend individuals in their network to invite.

One week prior to the meeting, the invited participants electronically received the BEFORE DA prototype. Participants also received a preliminary online survey administered through SurveyMonkey [52] that inquired about their preferred presentation of the statistics on the pregnancy success rates (pictographs or graphs) for each fertility option, format of the explicit values clarification method (i.e., pros/cons list, sliding scale, or Liket-scale), and if they believed an explicit values clarification method and personal stories should be included in the BEFORE DA.

The meeting was organized into three break-out group sessions focusing on different aspects of the BEFORE DA: (1) presentation of statistics; (2) inclusion and formatting of an explicit values clarification method; and (3a) inclusion and formatting of personal stories, (3b) dissemination strategies. Break-out groups included six to eight participants, with at minimum two patients/advocates per group and one reproductive specialist and medical/surgical oncologist per group. One designated note taker at each break-out group documented and facilitated the group discussion. Participants had the opportunity to first discuss the topics with their small break-out group, and then a representative from each group presented key points of the discussion to the larger group leading to an in-depth full group discussion on the topic [53]. Two designated note takers documented the full group discussion. All notes were compiled following the meeting and summarized to identify recommended modifications to the BEFORE DA.

### Step 3 – final prototype development (paper and online) and assessments

Our team revised the BEFORE DA prototype based on the meeting feedback. Meeting participants and individuals who were not able to attend the meeting were electronically sent the revised BEFORE DA to ensure the revisions accurately reflected the discussion at the meeting and that there was consensus among a wide range of stakeholders who did not participate in the meeting on the DA content. Using the BEFORE DA prototype, OfftoMarket Inc. [54] graphically designed it into a final paper DA and developed a mirrored online DA.

#### Health literacy

Prior to finalizing the paper prototype our team assessed its literacy level using the Suitability Assessment of Materials (SAM) [55] and the Flesh-Kincaid Grade Level [56]. The SAM assessment rates the readability and comprehension of educational materials through 21 questions across six sections allowing for 0 points (not suitable) to 2 points (superior) on each question for a possible total of 42 points. The SAM instrument evaluates six areas of materials including content, literacy demand, graphics, layout and type, learning stimulation and motivation, and cultural appropriateness [55]. The score of the 42 questions are totalled for an overall score which rates the material as a superior material (70 to 100%), an adequate material (40 to 69%), or not suitable material (0 to 39%) [57]. The assessment was completed by two independent reviewers and discrepancies were resolved by a separate reviewer. An online readability calculator [58] aided the Flesh-Kincaid Grade Level assessment. A patient education specialist also completed iterative reviews throughout the development process to ensure the BEFORE DA complied with plain language best practices [57, 59].

#### International patient decision aid standards criteria

The final BEFORE DA was assessed against the IPDAS checklist and instrument (v4.0), which contains six qualifying criteria for DAs, 10 certification criteria, and 28 quality criteria [60]. Two independent reviewers assessed the paper and online BEFORE DA against the IPDAS criteria with any discrepancies resolved by a separate reviewer.

### Step 4 – alpha testing

**Fig. 2:**
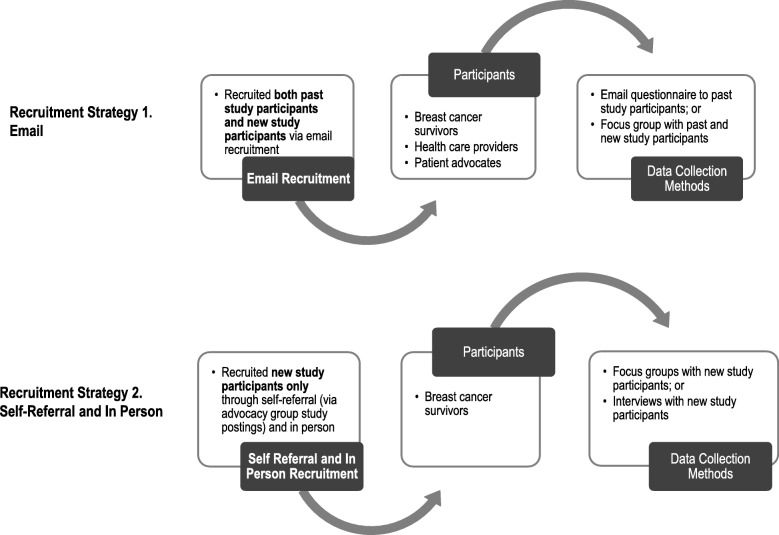
Recruitment strategy for alpha testing participants

The paper and online BEFORE DA underwent alpha testing to determine usability, acceptability, and comprehensibility. Participants included breast cancer survivors and HCPs with no previous involvement in the study as well as some who were previously involved in one or more of the preceding development steps (Fig. 2). New patient participants were recruited by self-referral through a recruitment poster on advocacy groups’ social media pages and in person at a breast cancer clinic in the Greater Toronto Area. New HCP participants were identified through the research teams’ professional networks and through snowball sampling [51] and were invited by email. New participants could participate in a focus group or a telephone interview if they could not attend the focus group. Past participants were invited by email to participate in the focus group. Past participants unable to attend the focus group were sent an email questionnaire on the BEFORE DA to complete and return at a time convenient with their schedules (Additional file 2).

Our team aimed to have at minimum 10 to 20 participants review the paper and online BEFORE DA as Faulkner [61] has demonstrated that 80% of usability problems are revealed with 10 participants, increasing to 95% with 20 participants. Recruitment occurred between April 2017 and May 2017, with alpha testing being conducted concurrently. The alpha testing guide (Additional file 3) included questions derived from The Ottawa Hospital Research Institute acceptability guide [62] and adapted usability questions developed by experts in qualitative research for a lung cancer screening DA [63]. Interviews were audio-recorded and extensive notes were taken during the focus group by two note takers. The key modifications recommended by participants during alpha testing were transcribed, thematically analyzed [64], and organized using Microsoft Excel 2010. Our team reviewed the recommendations to ensure no conflicts with past stakeholder recommendations prior to making the final revisions to the BEFORE DA.

## Results

The following paragraphs outline the results of the development process including the prototype development details, DA discussion and recommended DA modifications from the stakeholder meeting, designing of the final paper and online DA, and themes derived from the alpha testing that influenced final modifications in the DA.

### Step 1 – prototype development

Our team developed an 18-page paper prototype comprised of 11 sections: (1) about the DA (i.e., who the DA is for, what information is included in the DA, how to use the DA); (2) background information on fertility and the impact of breast cancer treatments on fertility; (3) most common fertility options (i.e., wait and see, embryo and oocyte cryopreservation, and ovarian suppression) with pregnancy success rates and risks in an option grid format separated by age; (4) common fertility questions before and after treatment; (5) parenthood options after cancer treatment; (6) timeline of when the fertility options are available; (7) question list for oncology and fertility HCPs; (8) sources for more information; (9) glossary; (10) team and acknowledgements; and (11) references.

Content experts’ revisions included clarification of information and emphasis on key messages (e.g., clarifying that fertility may still be compromised even if menstruation resumes post-treatment), re-formatting of the pregnancy success rate statistics for each fertility option (e.g., moving the statistics from the option grid to a separate section in the DA for clarity), and updating some information to reflect the most recent literature and Canadian practice.

### Step 2 – stakeholder engagement meeting

The one-day meeting occurred on November 11, 2016 in Toronto, Ontario with 28 multi-disciplinary stakeholders from across Canada (Table 1) and an experienced facilitator who was a general surgeon and medical decision-making expert. Of the 28 participants who were sent the DA prototype and survey, 23 (82%) provided responses. For the presentation of statistics, most respondents (61%) preferred the use of pictographs over graphs (30%). Almost all respondents (91%) felt an explicit values clarification method should be included in the DA. Finally, 70% of respondents felt that personal stories should be included in the DA.

**Table 1:** Demographic characteristics of participants who participated in the stakeholder engagement meeting (Step 2) and alpha testing (Step 4)

Demographic Characteristics	Stakeholder Engagement Meeting (*n* = 28)	Alpha Testing (*n* = 17)
Type of Stakeholder		
Reproductive Endocrinology and Fertility Specialist	4	1
General Surgeon (Specializing in Breast Cancer)	2	–
Medical Oncologist	3	3
Registered Nurse	2	–
Social Worker	1	–
General Practitioner	–	2
Decision Aid Expert	2	–
Patient Education Specialist	1	–
Breast Cancer Survivor	8	10
Cancer Organization and Advocate Representatives	5	–
Cancer Survivorship Expert	–	1
Location (All Stakeholders)		
Ontario	21	12
British Columbia	4	3
Québec	1	1
Alberta	1	–
Saskatchewan	–	1
Newfoundland	1	–
Hospital Setting (Health Care Providers)		
Community	3	3
Academic	9	4
Children Prior to Cancer Diagnosis (Survivors)		
Yes	**–**	3
No	**–**	6
Unknown (did not provide answer)	**–**	1
Relationship Status at Diagnosis (Survivors)		
Married	**–**	4
Long-Term Relationship	**–**	3
Single	**–**	2
Unknown (did not provide answer)	**–**	1
Self-Identified Ethnicity/ Race (Survivors)		
Filipino	**–**	1
Hispanic	**–**	1
East Indian	**–**	1
White	**–**	2
Persian/Iranian	**–**	1
African Canadian	**–**	1
Chinese/Portuguese	**–**	1
Unknown (did not provide answer)	**–**	2
Education (Survivors)		
Some College or Post High School Training	**–**	2
Post-Secondary	**–**	1
Graduate Level Studies/Professional Degree	**–**	5
Unknown (did not provide answer)	**–**	2
Age Range at Diagnosis (Survivors)	**–**	21 to 43

Meeting participants collaboratively discussed the presentation of statistics in the first break-out group. Some participants felt that while information portrayed using bar graphs may be easier to understand for some patients, graphs may not convey the information as clearly when compared to pictographs. However, survivor participants noted that pictographs may cause more anxiety in patients as it makes the pregnancy outcomes seem more real and it is difficult information to hear or read. Therefore, participants expressed that while best estimates should be presented for the pregnancy outcomes based on current knowledge the statistics should be presented with a message of hope that pregnancy is possible for some patients when any fertility option is chosen. Participants also emphasized the clarification of messaging when presenting statistics (e.g., the pregnancy rates for wait and see are based on the age the patient is attempting to get pregnant post-treatment compared to the pregnancy rates for embryo and oocyte cryopreservation which are based on the age the patient has oocyte retrieval). Overall, there was agreement that the BEFORE DA should present statistics for the fertility options success rates as pictographs, with the images of gender-neutral people for inclusivity and using a denominator of 10.

In the second break-out group, participants discussed the inclusion of an explicit values clarification method in the BEFORE DA and agreed that one should be included. Most participants preferred the example values clarification method adapted from a values clarification method in a DA from the Netherlands [14]. However, recommendations were made to revise the values clarification method by changing the headings to ‘important to me’ and ‘not important to me’, having a Likert-scale in the online DA, and modifying the pre-listed values to encompass more psychological aspects of care. Participants noted the value of including blank spaces after the pre-listed values for patients to include additional values they consider important.

The final breakout group focused on personal stories and dissemination strategies. Participants agreed that we should not be ‘recreating the wheel’ for personal stories and should alternatively provide links to organizations and sources already in existence within the BEFORE DA. While there was some concern among participants that patients in similar situations may decide on the same option as the person sharing their story, there was also acknowledgement among patients that they valued personal stories throughout their care. Participants recommended any included personal stories should show the good and bad of the fertility options and be modern. Overall, there was agreement on the inclusion of quotes from past breast cancer patients as ‘words of wisdom’ and a form of personal stories in the online DA. Additionally, the group identified a range of strategies for national and local dissemination of the DA. Table 2 outlines the final recommendations for the BEFORE DA from meeting participants.

**Table 2:** Stakeholder engagement meeting final recommendations for the BEFORE (Begin Exploring Fertility Options, Risks, and Expectations) decision aid

Break-out Group Session	Final Recommendations
1. Presentation of statistics	− Use pictographs for the visual presentation of statistics on the pregnancy success rates for each fertility option − Reduce the denominator from 100 to 10 − Use a gender-neutral image for the pictograph images to ensure inclusivity
2. Inclusion and formatting of an explicit values clarification method	− All participants felt that an explicit values clarification method should be included as an optional tool for those patients who want to use it − Most participants preferred the Likert-scale format with pre-listed values and blank spaces for patients to add in their own values − Suggested modifying the scale headings to ‘important to me’ and ‘not as important to me’ for the values
3a. Inclusion and formatting of personal stories	− Personal stories through videos, quotes, and forums were viewed as beneficial for inclusion − Caution was expressed on ‘recreating the wheel’ as many stories exist online currently − Diverse patients and modern stories were requested to ensure representation and a sense of personalization for the patient viewing the story − Recommendations to include ‘quotes of wisdom’ from breast cancer survivors throughout the decision aid
3b. Dissemination strategies	National/Broad Strategies − Determining a host location to reach as many young breast cancer patients as possible (e.g., national cancer organizations or advocacy groups) − National dissemination (e.g., providing links to the decision aid through national cancer organizations or advocacy groups) − Add to the curriculum for medical students as well as continuing medical education for health care providers already in practice − Advertise the decision aid in medical journals − Search engine optimization for the online decision aid Local Strategies − Flag use of the decision aid in electronic medical records − Include use of decision aid in physician checklists − Provide information and dissemination in general surgery updates, tumour boards, or multidisciplinary cancer conferences − Directed patient advertising (e.g., have posters on the walls of physician office) − Survivor and health care provider champions

### Step 3 – final prototype development (paper and online) and assessments

Following the meeting and subsequent review by meeting attendees and content experts not able to attend, the pregnancy success rate statistics for the fertility options were modified to include a table for each fertility option with the success rates broken down by age categories (e.g., under age 30, 30 to 34, 35 to 39, 40 to 44, over age 44). Limited information is available on pregnancy rates post-treatment for breast cancer patients using each of the fertility options by age. As such, the team used the best available evidence from the Canadian Fertility and Andrology Society (CFAS) [39] and the literature [37, 40–42, 65–68] to determine the estimated pregnancy success rates by age for wait and see, embryo cryopreservation, and ovarian suppression and had these rates confirmed by four reproductive specialists.

From the paper prototype to final paper and online BEFORE DA, our team used stakeholder feedback through the iterative reviews and the stakeholder meeting to revise and condense the existing DA sections and included two new sections: (1) a one-page summary and (2) information on fertility after breast cancer treatment. With the paper prototype reviewed through four iterative rounds with stakeholders, our team finalized the content and created a 32-page graphically designed paper DA containing 12 sections with images of diverse women used to separate each section of the DA. The paper DA can be downloaded by any user who accesses the online DA [69]. Using the final paper BEFORE DA, OfftoMarket Inc. [54] created a mirrored online version of the BEFORE DA that is accessible on any operating system [69]. Additional file 4 shows example pages of the paper BEFORE DA including the table of contents, pregnancy success rates for embryo freezing, timeline of fertility options, and part of the values clarification exercise. The full DA is available at https://fertilityaid.rethinkbreastcancer.com.

The online and paper BEFORE DA is available in English and French. Translation of the BEFORE DA from the original English version to French was completed by a certified translation company [70]. The translated DA was then verified by a health care professional and a member of the public fluent in French and English to ensure all concepts translated correctly from both a medical and plain language perspective. During the review, some concepts had to be revised as they had different meanings in each language. For example, ‘decision support tool’ was used in the French version of the DA and ‘decision aid’ is used in the English version. Additionally, the English version of the BEFORE DA contains gender neutral language, while the French version includes gender neutral language where possible and when it was not possible the feminine form is used (e.g., for the words ‘pregnant’ and ‘menopause’). The team worked with the translation company to ensure the translation was as inclusive as possible while still following the terminology referenced in French dictionaries at the time of the translation.

The online version of the DA contains the same sections as the paper version, in addition to supplementary sections stakeholders deemed valuable to include for patients who require more information and to avoid overwhelming patients with extra content in the paper DA (e.g., cost of FP and financial assistance separated by province and territory in Canada is only included in the online DA but the paper DA includes multiple references to the online DA to direct interested patients and HCPs). The online DA design guides users through the DA using a ‘next button’ at the bottom of each DA section. Alternatively, users are also able to directly access any DA section using the ‘section menu.’ A key feature of the online DA is a printable package that contains patient’s responses to the values clarification method and the HCP question list. The DA guides patients to complete and print this package for their clinical appointments to help facilitate fertility discussions with their care team. The online DA is also compliant with the Web Content Accessibility Guidelines [71] set out as part of the Accessibility for Ontarians with Disability Act (AODA). Table 3 describes the paper and online BEFORE DA sections and provides a brief description of each section.

**Table 3:** Description of sections in the BEFORE (Begin Exploring Fertility Options, Risks, and Expectations) decision aid

Sections	Section Details
About the decision aid	− Information on who the decision aid is for, what it includes, proper use, and that it does not replace medical information from health care providers
Background information	− Concise information on breast cancer treatments/age-related fertility decline − Potential fertility outcomes after treatment − Frequently asked questions on fertility before cancer treatment
Fertility options before treatment	− Information on the most common fertility options including an option grid comparing the different options − Pregnancy success rates after treatment with the most common fertility options depicted using pictographs − Frequently asked questions on the fertility options before treatment
Parenthood options after treatment	− Information on parenthood options after treatment − Frequently asked questions on the parenthood options
Timeline of your fertility options	− Timeline of fertility options available during the care journey
Summary	− One-page summary of key information in the decision aid
Fertility options exercise	− Likert-scale explicit values clarification method with pre-listed values and blank spaces to add additional values
Questions	− Explanation of the different health care providers’ roles − List of 10 common questions for health care providers
What’s next	− List of national resources for more information and support (e.g., financial support and peer to peer support)
Fertility after breast cancer	− Information on life and fertility after breast cancer − List of national resources for more information and support
List of terms	− Definitions for medical terms used in the decision aid
Sources and recognition	− Brief description of development team, conflicts of interest statement, funding information, and date of last update and future updates − Reference list
*Additional sections in online decision aid*	
PDF download	− Downloadable paper decision aid (English or French)
Less common and experimental fertility options	− Information on the less common and experimental fertility options including an option grid comparing the different options
Cost of fertility preservation by province	− Drop-down list showing the funding and cost of fertility preservation by province/territory in Canada based on fertility clinic publically posted fees
Province/territory specific resources	− Drop-down list of resources for more information by province/territory in Canada
Quiz myself section	− Five questions for individuals to quiz themselves on the decision aid content and three summary questions to facilitate understanding of next steps
Interactive fertility options exercise and summary page	− Explicit values clarification method that patients can complete and receive a printable summary page of their responses with the 10 common questions for health care providers to print and bring to appointments
Personal quotes	− Quotes from breast cancer patients who experienced fertility decision-making before treatment
Full team member list	− Full list of team members who developed the decision aid
Contact page	− Individuals can ask questions or provide feedback to the development team

#### Health literacy

The final BEFORE DA is evaluated at a grade seven reading level in line with the recommendations by the IPDAS [59]. The BEFORE DA scored 35 out of a possible 42 points (83%) when evaluated using the SAM assessment, classifying it as a superior material (Additional file 5).

#### IPDAS criteria

The BEFORE DA met all qualifying and applicable certification criteria set out by the IPDAS, and most quality criteria (Additional file 6).

### Step 4 – alpha testing

Among the 17 HCPs invited to participate, seven (41.2%) provided feedback (one by email questionnaire and six through telephone interviews). Of the 18 breast cancer survivors invited to participate, 10 (55.6%) provided feedback (three through telephone interviews, three in a focus group, and four by email questionnaire). Table 1 describes the demographic characteristics of the alpha testing participants.

Four themes were discerned resulting in modifications to the BEFORE DA including: (1) layout and graphics; (2) comprehensibility and acceptability of information; (3) usability; and (4) use and delivery in clinical practice. Table 4 outlines the modifications made to the BEFORE DA following alpha testing and illustrative quotes from participants leading to the revisions. Not all suggested modifications could be incorporated into the BEFORE DA as they did not comply with the AODA guidelines. For example, participants recommended including a timeline graphic similar to the paper DA in the online DA, however this change would have impeded on screen readers ability to accurately convert the webpage text into synthesized speech. Additionally, some comments conflicted with previous consensus from stakeholders. For example, two HCP participants noted that they did not feel the values clarification method would be beneficial to patients during fertility decision-making. However, since the inclusion of the values clarification method was agreed on by participants at the stakeholder meeting it was not removed from the DA.

**Table 4:** Themes discerned from the alpha testing, illustrative quotes from patient and provider participants and resulting modifications made to the BEFORE (Begin Exploring Fertility Options, Risks, and Expectations) decision aid

Theme	Modifications made	Illustrative quotes from participants
Layout and graphics	1. Modified two photos that showed women laughing and smiling to diverse couples 2. Modified a graph that showed fertility decline with chemotherapy to show only natural declines in fertility as a person ages. Also, the graph was adjusted to a larger size for easier viewing. 3. In the 'test myself section' of the online decision aid, the correct answers were highlighted 4. Darkened the colour of the national drop-down table row and separated it from the list to emphasize the row.	1a. In regards to family types, I don’t believe I saw couples. If couple are included, perhaps consider including both oppose sex relationships and same-sex relationships. (Email, Patient 07) 1b. “…they all look very happy to me but it is multi-cultural. I just felt that this is often not a topic that women smile about, they are terrified about this…” (Interview, HCP 03) 2. As for the graph, it looks like the chance for fertility by age 37 is absolutely zero [with chemotherapy] and that is definitely not true so I don’t think it’s acceptable in its current format. I’ve had several patients get pregnant after chemo in their late 30’s or even very early 40’s. (Email, HCP 06) 3. …feedback sentences on quiz page should be a different colour than the answers. (Email, Patient 07) 4. Emphasize the national drop-down menu in the drop-down table of resources. People go straight to their province and do not realize that there are additional resources in the national section (FG, Patients 01, 02, 03)
Comprehensibility and acceptability of information	1. Modified the resource list to accurately represent all supports provided by each group 2. Test myself questions were reviewed and modified	1. “The Canadian Breast Cancer Society is now part of the Canadian Cancer Society … somewhere just capturing the nature of peer to peer support…if you don’t mention the peer to peer support you are missing the flavor of what the Canadian Cancer Society does.” (Interview, HCP 04) 2. …I might add a couple more [questions] and ask some specific questions to make sure that the women understand. The correct/incorrect answers will give the health care team and indication of what they need to explain further/if this woman is ready to make a decision. (Email, Expert Content Reviewer)
	3. Modified complex terminology (e.g., ‘per embryo transfer’ was modified to ‘each time embryos are put into the womb’). Completed health literacy tests and the patient education specialist reviewed the decision aid 4. Modified icon arrays to show each fertility option in a separate table. Experts confirmed estimated pregnancy success rates for each option and disclaimers were highlighted	3. “… I find it is maybe a little bit too complex. I think it could be simplified a little bit…I think the language is written at too high a level.” (Interview, HCP 02) 4. “…I find I am a bit confused. And if I am confused I would think someone else is confused… it looks like in fact if you freeze your embryos you have more chance of getting pregnant by waiting and seeing than embryo freezing because that is what it looks like when you have them juxtaposed…” (Interview, HCP 04)
Usability	1. Emphasized the navigation buttons in the online version 2. Modified the title of the navigation buttons to be more reflective of content 3. Moved the section menu so it is more apparent to users 4. Emphasized the ‘Next’ button for each page 5. Emphasized hyperlinks to information on cost and experimental fertility options	1. The blue buttons at the top of the decision aid where patients could download the paper decision aid and access the values clarification method were not noticed until specifically directed to the buttons. (FG, Patients 01, 02, 03) 2. “I don’t know if care kit is the right term for [the values clarification method]…maybe a decision checklist or something. It is not a kit to help me actually move forward with my treatment.” (Interview, Patient 04) 3. [referring to the section menu] “…no I didn’t actually…I am just noticing that now.” (Patient, 04) 4. The “Next” button at the bottom of the page was hard to see due to the similarities in colour against the background (FG, Patients 01, 02, 03) 5. Emphasize hyperlinks to access more information (e.g., “less common and experimental fertility options” and “Cost of fertility preservation page.”) (FG, Patients 01, 02, 03)
Use and delivery in clinical practice	No modifications made to the BEFORE decision aid based on this theme	

*Abbreviations*: *FG*, focus group; *HCP*, health care provider

In general all participants found the length of the DA to be appropriate. Some participants stated that the paper DA may be too long but also discussed the importance of all the information included and that information needs will vary among each patient, resulting in no recommendations to remove any information. Overall, the layout was approved by participants and it was noted that the information was well-organized into sections. Participants also thought there was the right amount of information, although a few noted that some messages (e.g., age-related fertility decline), were repetitive but important to include. Most participants felt the information was balanced between the fertility options presented. Minor wording changes were made to the adoption parenthood option as one HCP participant felt that the wording may discourage patients from considering it as a plausible option following treatment.

The feasibility of the DA in clinical practice was also discussed, with most HCP participants open and enthusiastic about recommending and using the DA in their clinics. Breast cancer participants recognized the importance of the DA and evaluated it favourably, especially since fertility was not uniformly discussed with them prior to commencing cancer treatment. Health care provider participants recommended the development of a strategic implementation plan to ensure use of the BEFORE DA for premenopausal breast cancer patients and the need for strategies to include education for HCPs on oncofertility, the BEFORE DA, and referral pathways to reproductive specialists. The combination of a paper and online DA was also seen as useful since some patients prefer a paper take-away after clinic, while others prefer to access information online. To enable online access among premenopausal breast cancer patients, Rethink Breast Cancer [72], a charity that empowers young individuals diagnosed with breast cancer and addresses their unique needs, is hosting the BEFORE DA.

## Discussion

This paper details the development process and alpha testing for the BEFORE DA, an online and paper oncofertility DA for premenopausal breast cancer patients. Using an iKT approach, key stakeholders who will utilize the BEFORE DA in practice were engaged at the beginning of the development process and collaboratively worked with the research team to create the DA [23]. The use of iKT allowed for the development of a DA that addresses the information needs of breast cancer patients making FP decisions [6] as well as the approval and insights from multiple oncology and fertility HCPs who will be delivering and/or using the DA as an adjunct to their FP discussions with patients. By collaborating with multiple key stakeholders throughout the development of the BEFORE DA, we anticipate greater uptake and usability of the DA in clinical settings with premenopausal breast cancer patients making fertility decisions before treatment.

The majority of engaged stakeholders deemed a detailed DA as the appropriate format for the BEFORE DA. In the previous needs assessment interviews conducted by our team, a simple resource (e.g., an option grid) that relies on HCP delivery/use with patients in clinic to enhance shared decision-making [73] was viewed by breast cancer survivors as a useful but insufficient tool for their needs [27]. Further most DA development processes separate HCP and patient stakeholders when testing and reviewing DAs, however our team opted to bring critical stakeholders together through a one-day stakeholder meeting to facilitate collaborative discussions and ensure successful implementation and use of the BEFORE DA in clinical practice. By bringing these stakeholders together a fruitful discussion emerged between varying participants and agreement was reached on how to move forward with the design and content for inclusion in the final BEFORE DA.

At the time of the BEFORE DA development, oncofertility DAs were also available for Australian [13] and Dutch [14] premenopausal breast cancer patients. Our team utilized these existing DAs and modified the sections participants felt were valuable for inclusion in the BEFORE DA (e.g., the values clarification exercise was adapted from the Netherlands DA). While other fertility DAs and educational materials provide general information on the success rates of the fertility options, the BEFORE DA attempts to provide more personalized information through the presentation of estimated pregnancy success rates for the most common fertility options split into age categories. These rates are depicted using pictographs as recommended by the Ottawa Decision Support Tutorial [19]. The BEFORE DA also has unique components such as information and sections that are applicable to the Canadian population. (e.g., resources, cost of FP, and funding of FP are separated by province and territory in Canada). Additionally, the BEFORE DA is available as both a standalone paper DA (similar to the Australian DA) and online DA (similar to the Dutch DA). The BEFORE DA displays information on the most common fertility options before treatment in an option grid format, similar to an existing fertility option grid in Canada [74]. Common to DAs the BEFORE DA includes background information on fertility, the fertility outcomes after treatment and the options available. The BEFORE DA does not include general information on the risks to breast cancer in the population as stakeholders felt this information was not required during the time of fertility decision-making. Finally, the BEFORE DA is available in both English and French to ensure a wider range of patients will be able to utilize the tool if they want more fertility information following their cancer diagnosis. The development process is easily adaptable by other developers and the BEFORE DA content can be modified and adapted to other jurisdictions and to other tumour types based on the specific needs of those who will be utilizing the DA.

Our alpha testing results suggest that the BEFORE DA is usable and feasible in clinical practice. A strategic implementation plan was recommended that was inclusive of education for HCPs on oncofertility, the BEFORE DA, and referral pathways to reproductive specialists and other providers as needed. The BEFORE DA is one important piece to address the fertility information gaps following a breast cancer diagnosis [5]. As the BEFORE DA becomes widely available, education for HCPs is necessary to ensure the DA is successfully implemented in clinical practice and that fertility is discussed with premenopausal patients prior to treatment [75–77]. Future work to develop a HCP targeted tool would also be of value to complement the BEFORE DA. Our team aims to utilize recommendations from the stakeholders to disseminate information on oncofertility and the BEFORE DA through webinars with HCPs in field testing (Fig. 1) and by making the DA accessible on a prominent advocacy group’s webpage. As part of the agreed hosting responsibilities, Rethink Breast Cancer will ensure the content in the BEFORE DA is updated, including the pregnancy success rates with embryo and oocyte cryopreservation, risks to fertility from new treatment regimens, and fertility preservation options. Rethink Breast Cancer has a dedicated medical advisory group who will review the content of the BEFORE DA. Full reviews of the DA will occur every 2 years or when new information becomes available. The team will review the ASCO fertility guideline updates to determine new fertility options recommended and what treatments pose a risk to fertility. In addition, the annual CFAS reports will be reviewed to determine any change in pregnancy success rates from oocyte and embryo cryopreservation.

By partnering with an existing advocacy group that has devoted resources for innovative education and support, our team is confident in the sustainability of the BEFORE DA as an effective resource for premenopausal breast cancer patients and HCPs. Alternative strategies could also focus on integrating the BEFORE DA into electronic medical records through prompts, similar to work completed by Hand et al., who successfully embedded a fertility clinical decision support system in electronic medical records in an Australian paediatric hospital [78].

While the BEFORE DA was systematically developed following international standards, there are limitations to the process. Many stakeholders were involved in the development from all aspects of premenopausal breast cancer patients’ care except for patient’s partners or support person(s). These individuals were considered important members of the care team and influential in the decision-making process by patients [79, 80]. While we do not anticipate a major change to the design or content in the BEFORE DA with the inclusion of partners or support person(s), they may have provided additional insights or recommended a companion tool targeted to their specific information needs. Finding the balance of information for inclusion in DAs can be challenging [81] and while the BEFORE DA covers an array of information, patients’ information needs may extend past the content contained in the resource. Additionally, while all content in the DA was reviewed by multiple content experts, and came from reliable information sources and expert consensus, the BEFORE DA should only be used as an adjunct to clinical consultations; HCPs should still refer patients to reproductive specialists and provide tailored information to patients’ unique situation. Additionally, judgement against the IPDAS criteria was conducted by members of our team and should be validated through an independent third party not involved in the development. During alpha testing, the overall response rate was low and there were fewer than 10 participants for each unique stakeholder group. A self-selection bias may exist as the engaged stakeholders represent those who have a specific interest in fertility for cancer patients and those who did not respond may not have an interest in fertility. Despite this, 17 participants were recruited to evaluate the BEFORE DA and the key usability issues were reiterated by multiple participants allowing our team to have confidence that the main issues were discovered. While our team aimed to get representation from a wide variety of stakeholders, most patient participants had post- high school education. As such, the results on the usability and acceptability of the BEFORE DA may differ for individuals who are less educated. Additionally, even though the BEFORE DA was written at an acceptable reading level based on the IPDAS criteria, the current literature recommends patient education materials be written at a grade 5 level for patients with low literacy [82] and as such the BEFORE DA may not be suitable for all premenopausal breast cancer patients. However, to mitigate this concern our team completed plain language reviews throughout the development and completed multiple literacy tests to ensure readability and compliance with international standards.

## Conclusion

The BEFORE DA was created to fill the gap in fertility information needed for Canadian premenopausal breast cancer patients and can be easily modified for other jurisdictions. A range of stakeholders had an integral part in the development process and alpha testing. Health care providers expressed their interest in use of the DA in clinical practice and alpha testing participants provided an overall positive evaluation of the DA. The BEFORE DA aims to facilitate earlier fertility discussions between oncology HCPs and premenopausal breast cancer patients and, if appropriate, referrals to reproductive specialists, as well as assist patients in making informed value-based fertility decisions. Next steps for the BEFORE DA will focus on clinical implementation and testing the impact of the DA on oncofertility decision-making for newly diagnosed patients.

## Supplementary information

**Additional file 1.** Values clarification method options presented to stakeholder meeting attendees for feedback.

**Additional file 2.** Sample alpha testing email questionnaire to past study participants.

**Additional file 3.** Sample alpha testing interview and focus group questions.

**Additional file 4.** Sample pages from the paper BEFORE DA.

**Additional file 5.** Suitability Assessment of Materials (SAM) Assessment of the BEFORE DA.

**Additional file 6.** International Patient Decision Aid Standards Criteria Assessment of the BEFORE DA.

## Data Availability

The qualitative datasets generated during and/or analysed during the current study are not publicly available to maintain confidentiality and privacy for all participants.
