# Supplementary material for: The "Begin Exploring Fertility Options, Risks and Expectations" (BEFORE) decision aid: development and alpha testing of a fertility tool for premenopausal breast cancer patients
Source: BMC Med Inform Decis Mak. 2019 Oct 28;19:203. doi: 10.1186/s12911-019-0912-y (PMC6819618; doi:10.1186/s12911-019-0912-y)
Supplement: Supplementary file 1 — Additional file 1. Values clarification method options presented to stakeholder meeting attendees for feedback. [file 12911_2019_912_MOESM1_ESM.docx]

**Additional File 1.** Values clarification method options presented to stakeholder meeting attendees for feedback

# Explicit Value Clarification Methods – Examples

EXAMPLE 1 – Pro/Con Charts

**Options 1:** Pro/Con Charts with pre-filled information

**Adopted from:** Peate M, Meiser B, Friedlander M, Saunders C, Martinello R, Wakefield CE, et al. Development and Pilot Testing of a Fertility Decision Aid for Young Women Diagnosed with Early Breast Cancer. The Breast Journal. 2011;17:112-4

**Instructions:** This section will help you through the process of making a fertility decision before treatment by listing the pros and cons for each option. Please review all the pros and cons listed for each option and add your own pros and cons to the charts. After reviewing each chart, circle on the scale how you are feeling towards the fertility option. At the end consider all the pros and cons and circle the fertility option you are leaning towards overall.

## Wait and See

| **Categories** | **Pros** | **Cons** |
| --- | --- | --- |
| **Cost** | There is no cost | I may regret not spending the money to preserve my fertility if I become infertile after cancer treatment |
| **Time** | I will not have to wait to begin cancer treatment | I may be infertile after cancer treatment and unable to have a natural pregnancy |
| **Future Children** | If I become infertile I have the option to  adopt/foster children or use donor eggs | If I become infertile after treatment I will not be  able to have a biologically related child |
| **Other** - *Input other pros and cons you consider important* |  |  |

**Please circle on the scale what you are leaning towards for the Wait and See Option**

| I am leaning towards waiting to see | I am still not sure | I am NOT leaning towards waiting to see |
| --- | --- | --- |

## Embryo Freezing

| **Categories** | **Pros** | **Cons** |
| --- | --- | --- |
| **Cost** | I will spend the money and have stored embryos with my own eggs for future use | There may be a cost to freeze the embryos and ongoing costs to store the embryos  My cancer treatment may not cause infertility and I already spent money to preserve my embryos |
| **Time** | My treatment could be delayed by 2 to 4 weeks  *The extent of the delay depends on my specific circumstances* | My health care team may feel as though I do not have time to delay beginning my treatment |
| **Future Children** | Children will be genetically related to me and the individual who provided sperm | Sperm is required and can be obtained from my partner or donor  There is no guarantee that freezing embryos will result in a child after my cancer treatment |
| **Ethical Considerations** | Before embryo freezing, I must decide what to do with the embryos if I no longer need them  *This decision can be made with my partner and health care team* | If I use sperm from my partner, we both must decide what to do with the embryos if we do not require them anymore |
| **Other** - *Input other pros and cons you consider important* |  | The hormones I get to collect my eggs may lead to ovarian hyper stimulation syndrome |

**Please circle on the scale what you are leaning towards for the Embryo Freezing Option**

| I am leaning towards embryo freezing | I am still not sure | I am NOT leaning towards embryo freezing |
| --- | --- | --- |

## Egg Freezing

| **Categories** | **Pros** | **Cons** |
| --- | --- | --- |
| **Cost** | I will spend the money and have my own eggs stored for future use | There may be a cost to freeze my eggs and ongoing costs to store my eggs  My cancer treatment may not cause infertility and I already spent money to preserve my eggs |
| **Time** | My treatment could be delayed by 2 to 4 weeks  *The extent of the delay depends on my specific circumstances* | My health care team may feel as though I do not have time to delay beginning my treatment |
| **Future Children** | Sperm is not required at the time of freezing Children will be genetically related to me | Egg freezing has lower live birth success rates than embryo freezing  There is no guarantee that freezing my eggs will result in a child after cancer treatment |
| **Other** - *Input other pros and cons you consider important* |  | The hormones I get to collect my eggs may lead to ovarian hyper stimulation syndrome |

**Please circle on the scale what you are leaning towards for the Egg Freezing Option**

| I am leaning towards egg freezing | I am still not sure | I am NOT leaning towards egg freezing |
| --- | --- | --- |

## Ovarian Suppression

| **Categories** | **Pros** | **Cons** |
| --- | --- | --- |
| **Cost** | There may be a cost that is less than embryo or egg freezing | The costs can add up over many months based on how long I am receiving chemotherapy  *The cost will depend on my insurance drug plan* |
| **Time** | I will not have to wait to begin cancer treatment | I will need to be on it throughout chemotherapy |
| **Future Children** | If it is successful I will have children that are genetically related to me  I can use it with other fertility options include embryo and egg freezing | If it does not work for me, I may be infertile after cancer treatment and unable to have a natural pregnancy |
| **Other** - *Input other pros and cons you consider important* |  | I may experience menopausal type feelings and/or osteopenia/ osteoporosis |

**Please circle on the scale what you are leaning towards for the Ovarian Suppression Option**

| I am leaning towards ovarian suppression | I am still not sure | I am NOT leaning towards ovarian suppression |
| --- | --- | --- |

### Which option am I leaning towards?

**Thinking about your pros and cons list circle which fertility option you are leaning towards overall.**

| Wait and See | Embryo Freezing | Egg Freezing | Ovarian Suppression | Still Not Sure |
| --- | --- | --- | --- | --- |

**Option 2:** Blank Pro/Con Chart

**Adapted from:** Fortnum D, Smolonogov T, Walker R, Kairaitis L, Pugh D. ‘My kidneys, my choice, decision aid’: supporting shared decision making. Journal of renal care. 2015;41:81-7.

**Instructions:** This section will help you through the process of making a fertility decision. Please take some time to think about the pros and cons for each of the fertility options in this decision aid and fill in the chart. At the end consider all pros and cons for each option and circle the fertility option you are leaning towards overall.

| **Fertility Option** | **Pros** | **Cons** | **Additional Thoughts** |
| --- | --- | --- | --- |
| **Wait and See** |  |  |  |
| **Embryo Freezing** |  |  |  |
| **Egg Freezing** |  |  |  |
| **Ovarian Suppression** |  |  |  |

### Which option am I leaning towards?

**Thinking about your pros and cons list circle which fertility option you are leaning towards overall.**

| Wait and See | Embryo Freezing | Egg Freezing | Ovarian Suppression | Still Not Sure |
| --- | --- | --- | --- | --- |

EXAMPLE 2 – Sliding Scale

**Adopted from:** Garvelink MM, ter Kuile MM, Fischer MJ, Louwé LA, Hilders CGJM, Kroep JR, et al. Development of a Decision Aid about fertility preservation for women with breast cancer in the Netherlands. Journal of Psychosomatic Obstetrics & Gynecology. 2013;34:170-8.

**Instructions:** This section will help you through the process of making a decision by rating the different options. Please go through each fertility option and check √ if you think each factor is a disadvantage or advantage. There is also space to fill in other factors that are important to you. The fertility option with the most check marks on the advantage side may be the best option for you. At the end consider all the options and circle the fertility option you are leaning towards overall.

| **Wait and See** | **This is a disadvantage** | |  |  | **This is an advantage** | |
| --- | --- | --- | --- | --- | --- | --- |
| There is no cost |  |  |  |  |  |  |
| I will not have to wait to begin cancer treatment |  |  |  |  |  |  |
| If I become infertile I have the option to adopt/foster children or donor eggs |  |  |  |  |  |  |
| I may be infertile after cancer treatment and unable to have a natural  pregnancy |  |  |  |  |  |  |
| Other: |  |  |  |  |  |  |
| Other: |  |  |  |  |  |  |
| **Embryo Freezing** | **This is a disadvantage** | |  |  | **This is an advantage** | |
| There may be cost to freeze embryos  and ongoing costs to store my embryos |  |  |  |  |  |  |
| My cancer treatment may not cause  infertility and I already spent money to preserve my embryos |  |  |  |  |  |  |
| I will have stored embryos with my  own eggs for future use |  |  |  |  |  |  |
| My treatment could be delayed by 2  to 4 weeks depending on my specific circumstance |  |  |  |  |  |  |
| Children will be genetically related to  me and the individual who provided sperm |  |  |  |  |  |  |
| Sperm is required and can be obtained  from a partner or donor |  |  |  |  |  |  |
| There is no guarantee that freezing embryos will result in a child that is  related to me |  |  |  |  |  |  |
| If I use sperm from my partner, we both must decide what to do with the embryos if we do not require them  anymore |  |  |  |  |  |  |
| Other: |  |  |  |  |  |  |
| Other: |  |  |  |  |  |  |

| **Egg Freezing** | **This is a disadvantage** | |  |  | **This is an advantage** | |
| --- | --- | --- | --- | --- | --- | --- |
| There may be a cost to freeze my eggs  and ongoing costs to store my eggs |  |  |  |  |  |  |
| My cancer treatment may not cause  infertility and I already spent money to preserve my eggs |  |  |  |  |  |  |
| I will have my own eggs stored for  future use |  |  |  |  |  |  |
| My treatment could be delayed by 2 to 4 weeks depending on my specific  circumstance |  |  |  |  |  |  |
| Sperm is not required at the time of  my egg freezing |  |  |  |  |  |  |
| Egg freezing has lower live birth  success rates than embryo freezing |  |  |  |  |  |  |
| Children will be genetically related to  me |  |  |  |  |  |  |
| There is no guarantee that freezing  my eggs will result in a child after cancer treatment |  |  |  |  |  |  |
| Other: |  |  |  |  |  |  |
| Other: |  |  |  |  |  |  |
| **Ovarian Suppression** | **This is a disadvantage** | |  |  | **This is an advantage** | |
| There may be a cost that is less than  embryo or egg freezing |  |  |  |  |  |  |
| The costs can add up over many months based on how long I am receiving chemotherapy  *The cost will depend on my insurance drug plan* |  |  |  |  |  |  |
| I will not have to wait to begin cancer  treatment |  |  |  |  |  |  |
| If it is successful I could have  children that are genetically related to me |  |  |  |  |  |  |
| If it does not work for me, I may become infertile and unable to have a  natural pregnancy |  |  |  |  |  |  |
| I can use it with other fertility options  include embryo and egg freezing |  |  |  |  |  |  |
| Other: |  |  |  |  |  |  |
| Other: |  |  |  |  |  |  |

### Which option am I leaning towards?

**Thinking about your pros and cons list circle which fertility option you are leaning towards overall.**

| Wait and See | Embryo Freezing | Egg Freezing | Ovarian Suppression | Still Not Sure |
| --- | --- | --- | --- | --- |

EXAMPLE 3 – Rating Scale

**Adopted from:** Metcalfe K, Poll A, O’connor A, Gershman S, Armel S, Finch A, et al. Development and testing of a decision aid for breast cancer

prevention for women with a BRCA1 or BRCA2 mutation. Clinical genetics. 2007;72:208-17

O'Connor A, Stacey D, Boland L. Ottawa Decision Support Tutorial. 2015. https://decisionaid.ohri.ca/odst/pdfs/odst.pdf. Accessed 12 June 2016.

**Instructions:** This section will help you through the process of making a decision by rating the different options. Please rate each factor presented for the options and include any other factors that are important for you. The fertility option that has the most number 4 and 5 (important to most important) in the *Reasons to CHOOSE this fertility option* column may be the best option for you. At the end consider all the options and circle the fertility option you are leaning towards overall.

| **Fertility Options** | **Reasons to CHOOSE this fertility option** | **How important is it to you?**  **0= Not important 5=Very Important** | | | | | | **Reasons to DECLINE this fertility option** | **How important is it to you?**  **0= Not important 5=VeryImportant** | | | | | |
| --- | --- | --- | --- | --- | --- | --- | --- | --- | --- | --- | --- | --- | --- | --- |
| Wait and See | There is no cost | 0 | 1 | 2 | 3 | 4 | 5 | I may regret not spending the money to preserve my fertility if I become infertile after cancer  treatment | 0 | 1 | 2 | 3 | 4 | 5 |
|  | I will not have to wait to begin  cancer treatment | 0 | 1 | 2 | 3 | 4 | 5 | I may be infertile after cancer  treatment and unable to have a natural pregnancy | 0 | 1 | 2 | 3 | 4 | 5 |
|  | If I become infertile I have the  option to adopt/foster children or use donor eggs | 0 | 1 | 2 | 3 | 4 | 5 | If I become infertile after  treatment, I will not be able to have a biologically related child | 0 | 1 | 2 | 3 | 4 | 5 |
|  | Other Reasons: | 0 | 1 | 2 | 3 | 4 | 5 | Other Reasons: | 0 | 1 | 2 | 3 | 4 | 5 |
|  |  | 0 | 1 | 2 | 3 | 4 | 5 |  | 0 | 1 | 2 | 3 | 4 | 5 |
| Embryo Freezing | I will spend the money and have stored embryos with my own eggs for future use | 0 | 1 | 2 | 3 | 4 | 5 | There may be a cost to freeze my embryos and ongoing costs to store  my embryos | 0 | 1 | 2 | 3 | 4 | 5 |
|  |  |  |  |  |  |  |  | My cancer treatment may not cause infertility and I already spent  money to preserve my embryos | 0 | 1 | 2 | 3 | 4 | 5 |
|  | My treatment could be delayed by 2 to 4 weeks.  *The extent of the delay depends on my specific circumstance* | 0 | 1 | 2 | 3 | 4 | 5 | My health care team may feel as though I do not have time to delay beginning my treatment | 0 | 1 | 2 | 3 | 4 | 5 |
|  | Children will be genetically related to me and the individual who provided sperm | 0 | 1 | 2 | 3 | 4 | 5 | Sperm is required and can be  obtained from my partner or donor | 0 | 1 | 2 | 3 | 4 | 5 |
|  |  |  |  |  |  |  |  | There is no guarantee that freezing  embryos will result in a child after my cancer treatment | 0 | 1 | 2 | 3 | 4 | 5 |
|  | Before embryo freezing, I must decide what to do with the embryos if I no longer need them.  *This decision can be made with your partner and health care team* | 0 | 1 | 2 | 3 | 4 | 5 | If I use sperm from my partner, we both must decide what to do with  the embryos if we do not require them anymore | 0 | 1 | 2 | 3 | 4 | 5 |
|  |  |  |  |  |  |  |  | The hormones I get to collect my eggs may lead to ovarian hyper  stimulation syndrome | 0 | 1 | 2 | 3 | 4 | 5 |
|  | Other Reasons: | 0 | 1 | 2 | 3 | 4 | 5 | Other Reasons: | 0 | 1 | 2 | 3 | 4 | 5 |
|  |  | 0 | 1 | 2 | 3 | 4 | 5 |  | 0 | 1 | 2 | 3 | 4 | 5 |

| **Fertility Options** | **Reasons to CHOOSE this fertility option** | **How important is it to you?**  **0= Not important 5=Very Important** | | | | | | **Reasons to DECLINE this fertility option** | **How important is it to you?**  **0= Not important 5=VeryImportant** | | | | | |
| --- | --- | --- | --- | --- | --- | --- | --- | --- | --- | --- | --- | --- | --- | --- |
| Egg Freezing | I will spend the money and have my own eggs stored for future use | 0 | 1 | 2 | 3 | 4 | 5 | There may be a cost to freeze my eggs and ongoing costs to store my  eggs | 0 | 1 | 2 | 3 | 4 | 5 |
|  |  |  |  |  |  |  |  | My cancer treatment may not  cause infertility and I already spent money to preserve my eggs | 0 | 1 | 2 | 3 | 4 | 5 |
|  | My treatment could be delayed  by 2 to 4 weeks.  *The extent of the delay depends on my specific circumstances* | 0 | 1 | 2 | 3 | 4 | 5 | My health care team may feel as  though I do not have time to delay beginning my treatment | 0 | 1 | 2 | 3 | 4 | 5 |
|  | Sperm is not required at the time  of freezing | 0 | 1 | 2 | 3 | 4 | 5 | Egg freezing has lower live birth  success rates than embryo freezing | 0 | 1 | 2 | 3 | 4 | 5 |
|  |  |  |  |  |  |  |  | The hormones I get to collect my  eggs may lead to ovarian hyper stimulation syndrome | 0 | 1 | 2 | 3 | 4 | 5 |
|  | Children will be genetically  related to me | 0 | 1 | 2 | 3 | 4 | 5 | There is no guarantee that freezing  my eggs will result in a child after cancer treatment | 0 | 1 | 2 | 3 | 4 | 5 |
|  | Other Reasons: | 0 | 1 | 2 | 3 | 4 | 5 | Other Reasons: | 0 | 1 | 2 | 3 | 4 | 5 |
|  |  | 0 | 1 | 2 | 3 | 4 | 5 |  | 0 | 1 | 2 | 3 | 4 | 5 |
| Ovarian Suppression | There may be a cost that is less than embryo or egg freezing | 0 | 1 | 2 | 3 | 4 | 5 | The costs can add up over many months and will depend on how long I am receiving chemotherapy  *The cost will depend on my insurance drug plan* | 0 | 1 | 2 | 3 | 4 | 5 |
|  | I will not have to wait to begin  cancer treatment | 0 | 1 | 2 | 3 | 4 | 5 | I will need to be on it throughout  chemotherapy | 0 | 1 | 2 | 3 | 4 | 5 |
|  | If it is successful I will have children that are genetically related to me | 0 | 1 | 2 | 3 | 4 | 5 | If it does not work for me, I may be infertile after cancer treatment and unable to have a natural  pregnancy | 0 | 1 | 2 | 3 | 4 | 5 |
|  | I can use it with other fertility options include embryo and egg  freezing | 0 | 1 | 2 | 3 | 4 | 5 | I may experience menopausal type feelings and/or osteopenia/  osteoporosis | 0 | 1 | 2 | 3 | 4 | 5 |
|  | Other Reasons: | 0 | 1 | 2 | 3 | 4 | 5 | Other Reasons: | 0 | 1 | 2 | 3 | 4 | 5 |
|  |  | 0 | 1 | 2 | 3 | 4 | 5 |  | 0 | 1 | 2 | 3 | 4 | 5 |

### Which option am I leaning towards?

**Thinking about what is most important to you circle which fertility option you are leaning towards.**

| Wait and See | Embryo Freezing | Egg Freezing | Ovarian Suppression | Still Not Sure |
| --- | --- | --- | --- | --- |

Example 4 – Blank Rating Scale

**Instructions:** This section will help you through the process of making a decision by rating your values. Please use this blank table and rating scale to identify values that you consider a priority. Rate the importance of each value on a scale from 0 (Not Important) to 5 (Very Important). This task will act as a visualization of values that are important to you and help to identify the fertility options that align with your values.

| **Most important values to me** | **How important is it to you? 0 = Not Important**  **5 = Very Important** | | | | | | **Which fertility option aligns with my values?**  Wait and See, Embryo Freezing, Egg Freezing, Ovarian Suppression |
| --- | --- | --- | --- | --- | --- | --- | --- |
| 1. *Example, having biological children is a priority for me.* | 0 | 1 | 2 | 3 | 4 | 5 | *Egg or Embryo Freezing* |
| 2. | 0 | 1 | 2 | 3 | 4 | 5 |  |
| 3. | 0 | 1 | 2 | 3 | 4 | 5 |  |

### Which option am I leaning towards?

**Thinking about what is most important to you circle which fertility option you are leaning towards.**

| Wait and See | Embryo Freezing | Egg Freezing | Ovarian Suppression | Still Not Sure |
| --- | --- | --- | --- | --- |
