# Supplementary material for: The "Begin Exploring Fertility Options, Risks and Expectations" (BEFORE) decision aid: development and alpha testing of a fertility tool for premenopausal breast cancer patients
Source: BMC Med Inform Decis Mak. 2019 Oct 28;19:203. doi: 10.1186/s12911-019-0912-y (PMC6819618; doi:10.1186/s12911-019-0912-y)
Supplement: Supplementary file 2 — Additional file 2. Sample alpha testing email questionnaire to past study participants. [file 12911_2019_912_MOESM2_ESM.docx]

**Additional File 2.** Sample alpha testing email questionnaire to past study participants

**BEFORE** Decision Aid - A decision **aid for young**  **breast cancer**

**patients in Canada**

My thoughts on the decision aid for fertility decision making after a breast cancer diagnosis

We would like to know what you think about the decision aid you have just received.

# Paper Decision Aid

1. Please rate each section, by checking ‘poor’, ‘fair’, ‘good’, or ‘excellent’ to show what you think about the way the information was presented on:

| About the decision aid pg. 3-4 |  | poor |  | fair |  | good |  | excellent |
| --- | --- | --- | --- | --- | --- | --- | --- | --- |
| Background pg. 5-8 |  | poor |  | fair |  | good |  | excellent |
| Fertility options before cancer treatment pg. 9-15 |  | poor |  | fair |  | good |  | excellent |
| Parenthood options after cancer treatment pg. 16-17 |  | poor |  | fair |  | good |  | excellent |
| Timeline of your fertility options pg. 18-19 |  | poor |  | fair |  | good |  | excellent |
| Summary pg. 20-21 |  | poor |  | fair |  | good |  | excellent |
| Fertility options exercise pg. 22-26 |  | poor |  | fair |  | good |  | excellent |
| Question List pg. 27-29 |  | poor |  | fair |  | good |  | excellent |
| What's Next? pg. 30 |  | poor |  | fair |  | good |  | excellent |
| Life after breast cancer pg. 31-32 |  | poor |  | fair |  | good |  | excellent |
| List of terms pg.33-34 |  | poor |  | fair |  | good |  | excellent |
| Recognition and sources pg. 35-36 |  | poor |  | fair |  | good |  | excellent |

Comments:

1. The length of the decision aid was *(check one)*:

|  |
| --- |
|  |
|  |

too long

too short

just right Comments:

**Additional File 2.** Sample alpha testing email questionnaire to past study participants (Continued)

1. The amount of information was *(check one)*:

|  |
| --- |
|  |
|  |

too much information

too little information just right

Comments:

1. I found the presentation *(check one)*:

|  |
| --- |
|  |
|  |

slanted towards wait and see

slanted towards doing fertility preservation

balanced

Comments:

1. This decision aid is a reliable method of helping patients make decisions about fertility before treatment *(check one)*:

Yes

No

Comments:

1. This strategy compliments my usual approach (*check one*):

Yes

No

Comments:

1. I found the decision aid *(check all that apply)*:

culturally appropriate

gender neutral

inclusive to all sexualities and family types (i.e., oppose sex relationships and same-sex relationships)

If you did not check one, please state your reason why:

**Additional File 2.** Sample alpha testing email questionnaire to past study participants (Continued)

# Online Decision Aid

1. How did you feel about the presentation of the web-based decision aid (e.g., layout, ability to navigate through sections, design, quotes)?
2. How did you find the values clarification exercise and summary page to print (under the "Care Kit") (e.g., any missing values or information in the summary page, ease of use of the summary page in clinic)?

# General Suggestions/Comments

1. What suggestions do you have to improve the paper and/or online decision aid?
2. Any other comments on the paper and/or online decision aid?

Thank you very much for taking the time to evaluate the Canadian fertility decision aid.

# References

O’Connor AM & Cranney A. User Manual – Acceptability [document on the internet]. Ottawa: Ottawa Hospital Research Institute; © 1996 [modified 2002; cited 2016 09 26] 5p. Available from https://decisionaid.ohri.ca/docs/develop/User_Manuals/UM_Acceptability.pdf
