# Supplementary material for: The "Begin Exploring Fertility Options, Risks and Expectations" (BEFORE) decision aid: development and alpha testing of a fertility tool for premenopausal breast cancer patients
Source: BMC Med Inform Decis Mak. 2019 Oct 28;19:203. doi: 10.1186/s12911-019-0912-y (PMC6819618; doi:10.1186/s12911-019-0912-y)
Supplement: Supplementary file 3 — Additional file 3. Sample alpha testing interview and focus group questions. [file 12911_2019_912_MOESM3_ESM.docx]

**Additional File 3**. Sample alpha testing interview and focus group questions

| **Questions** | **Additional Questions for Discussion** |
| --- | --- |
| **Presentation of the decision aid**   1. How did you feel about the presentation of the decision aid (online and paper)? | Paper and Online - Do you like the layout?  Paper and Online - Do you like the design? Added or removed?  Paper and Online - Did you find the decision aid culturally appropriate, gender neutral, and inclusive to all family types (e.g., oppose sex relationships and same-sex relationships)?  Online - Did you find it easy to navigate throughout the pages? How could it be made better?  Online - Did you need help navigating through the pages?  Online - Did you like the personal quotes used? Why or why not? |
| **Content in the decision aid**   1. What were your thoughts on the content in the decision aid? 2. Where there any areas of concern when you were reading the decision aid? | Paper and Online - Did you think the decision aid was too long, too short or just right? If too long, what sections do you think could be condensed?  Paper and Online - Did you think there was too much information, too little information or just the right amount of information in the decision aid?  Paper and Online - Did you find the presentation of information slanted to any fertility option? If so, which one and why?  Paper and Online - Is there any wording changes you think should be made to make the decision aid easier to read? |
| **Values clarification method**   1. How did you find the values clarification method and summary page to print? | Paper and Online - What is your impression of the summary of the values clarification questions?  Paper and Online - Are there any values you think are currently missing from the exercise?  Online - What are your thoughts on the format of the summary print document to take to the doctor’s appointment? Do you think any important information you may have wanted to have handy is missing? |
| **Accessing the decision aid**   1. How did you initially access the decision aid when it was sent to you? | Online - Phone, tablet, laptop or computer?  Online - Did you find it easy to access on the device you used?  Online - How could the access be improved if you had any difficulties? |
