# Supplementary material for: The "Begin Exploring Fertility Options, Risks and Expectations" (BEFORE) decision aid: development and alpha testing of a fertility tool for premenopausal breast cancer patients
Source: BMC Med Inform Decis Mak. 2019 Oct 28;19:203. doi: 10.1186/s12911-019-0912-y (PMC6819618; doi:10.1186/s12911-019-0912-y)
Supplement: Supplementary file 4 — Additional file 4. Sample pages from the paper BEFORE DA. [file 12911_2019_912_MOESM4_ESM.pdf]

Additional File 4. Sample pages from the paper BEFORE DA

BEFORE
Begin Exploring Fertility Options, Risks & Expectations

Table of Contents

About the Decision Aid 3

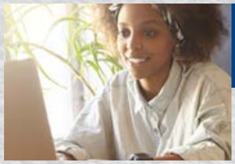

Section 1

Background information. 5

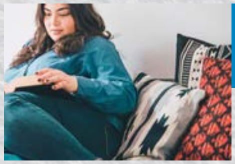

Section 2

Fertility options before treatment 9
Parenthood options after cancer treatment. 16
Timeline of your fertility options 17

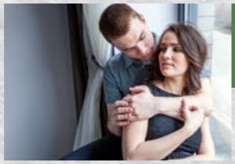

Section 3

Summary 19
Fertility options exercise 20

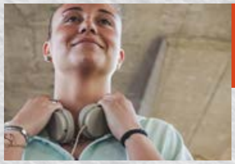

Section 4

Questions 25
What's next 27

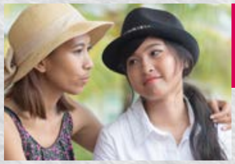

Section 5

Fertility after breast cancer 29

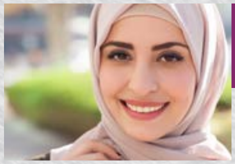

Section 6

List of terms 31
Sources and recognition 32

Embryo Freezing

What are my chances of pregnancy if I choose to freeze embryos before chemotherapy?

Your chance of having a pregnancy with embryo freezing is on top of any natural ability you may have to get pregnant after chemotherapy

The table below shows your chances of having a pregnancy by age range if you decide to freeze embryos. Remember that the chance of having a pregnancy in the general population under the age of 35 is only around 20% to 30% each month.

Age range when you freeze your embryos	Chance of having a pregnancy each time embryos are put into the womb
Under age 30 when you freeze embryos	Approximately **5 out of 10 people (50%)** will have a pregnancy each time embryos are put into the womb
30 to 34 years of age when you freeze embryos	Approximately **4 out of 10 people (40%)** will have a pregnancy each time embryos are put into the womb
35 to 39 years of age when you freeze embryos	Approximately **3 out of 10 people (30%)** will have a pregnancy each time embryos are put into the womb
40 to 44 years of age when you freeze embryos	Approximately **1 out of 10 people (10%)** will have a pregnancy each time embryos are put into the womb
Over age 44 when you freeze embryos	Your chances of having a pregnancy using your own eggs are very low. They are **less than 2%**

There are increased risks for any person who gets pregnant and has a child at an older age. Visit the Society of Obstetricians and Gynecologists in Canada for more information on the risks: www.pregnancy.sogc.org/fertility-and-reproduction/age-and-fertility/

Please note: A successful pregnancy will depend more on the age when your embryos were frozen before treatment.

Timeline of your fertility options

BEFORE
Begin Exploring Fertility Options, Risks & Expectations

The fertility options available to you depend on where you are in your cancer journey

1. Breast cancer diagnosis

Before you begin chemotherapy or hormone therapy all the fertility options may be available to you.

Fertility options:
- Wait and see
- Embryo freezing
- Egg freezing
- Ovarian suppression

2. Making a decision before chemotherapy and hormone therapy

Think about what is important to you for each fertility option. Talk with your partner or support persons and healthcare team to make a decision that is best for you.

Fertility options:
- Wait and see
- Embryo freezing
- Egg freezing
- Ovarian suppression

3. During chemotherapy and hormone therapy

Once you start treatment you are at risk of not being able to have a natural pregnancy. You cannot freeze your eggs or embryos at this time and should not get pregnant.

Fertility options:
- Wait and see

4. Making a decision after chemotherapy and hormone therapy

After treatment you may experience normal fertility, early menopause, or be unable to have a natural pregnancy even if your period returns

Fertility options:
- Natural pregnancy if your ovarian function returns
- In vitro fertilization and embryo transfer if you froze embryos or eggs before treatment or have high quality eggs remaining in your ovaries
- Egg or embryo donor
- Surrogacy
- Adoption

Fertility options exercise

BEFORE
Begin Exploring Fertility Options, Risks & Expectations

This exercise will help you think about what is important to you for each fertility option.

1. Go through each fertility option and check ✓ how important the listed factors are to you
2. Add in any other factors that are important to you

Making a decision on family planning can be emotionally difficult. Consider filling this out with a member of your healthcare team that you trust, with your partner or support persons.

Please note: you can decide on more than one fertility option. You do not have to make your fertility decision after completing this exercise. The exercise is also available for you to complete online at **fertilityaid.rethinkbreastcancer.com.**

Wait and See	Less Important to Me				More Important to Me
Example selection			✓		
There is no cost					
I can start my cancer treatment right away					
I do not need to inject myself with hormones					
I have other parenthood options available to me after treatment					
I do not need additional visits to the fertility clinic					
This process may add to my emotional stress					
I may be unable to have a natural pregnancy after treatment and may regret not keeping my options open					
Other:					

Remove this page from your decision aid and bring it to your medical appointments to discuss with your health care providers.
