# Supplementary material for: The "Begin Exploring Fertility Options, Risks and Expectations" (BEFORE) decision aid: development and alpha testing of a fertility tool for premenopausal breast cancer patients
Source: BMC Med Inform Decis Mak. 2019 Oct 28;19:203. doi: 10.1186/s12911-019-0912-y (PMC6819618; doi:10.1186/s12911-019-0912-y)
Supplement: Supplementary file 5 — Additional file 5. Suitability Assessment of Materials (SAM) Assessment of the BEFORE DA. [file 12911_2019_912_MOESM5_ESM.docx]

**Additional File 5.** Suitability Assessment of Materials (SAM) Assessment of the BEFORE (Begin Exploring Fertility Options, Risks, and Expectations) decision aid

| **SAM Quality Criteria** | **Rating and Description** | | |
| --- | --- | --- | --- |
| **Content** | | | |
| Purpose | | *Superior* | Purpose is explicitly stated in the title, cover illustration or introduction |
| Content Topics | | *Superior* | Thrust of the material is application of knowledge aimed at desirable reader behavior |
| Summary & Review | | *Superior* | Summaries are included and retell key messages in different words or examples |
| **Literacy Demand** | | | |
| Reading Grade Level | | *Adequate* | 6th to 8th grade |
| Writing Style | | *Superior* | 1) Conversational style and active voice are used throughout; 2) Simple sentences are used extensively |
| Sentence Construction | | *Superior* | Consistently provides context before presenting new information |
| Vocabulary | | *Superior* | All three factors: 1) common words are used all the time; 2) Technical, concept, category, value judgment words (CCVJ) are explained; 3) Appropriate imagery words are used |
| Learning Enhanced by Advance Organizers | | *Superior* | Nearly all topics are preceded by an advance organizer (a statement that tells what is next) |
| **Graphic Illustrations, Lists, Tables, Charts** | | | |
| Cover Graphics | | *Superior* | The cover graphic:1) Is friendly; 2) Attracts attention; and 3) Clearly portrays the purpose of the materials |
| Type of Illustration | | *Adequate* | One of the superior factors is missing  1) Simple adult-appropriate line drawings/sketches are used; 2) Illustrations are likely to be familiar to readers |
| Relevance of Illustrations | | *Adequate* | 1) Illustrations include some distractions; 2) Insufficient use of illustrations |
| Graphics: Lists, tables, charts, forms | | *Superior* | Provides step-by-step directions with an example that will build self-efficacy (confidence) |
| Captions are used to “announce” or explain graphics | | *Adequate* | Brief captions are used for some graphics |
| **Layout and Typography** | | | |
| Typography | | *Superior* | At least 3 of the following 4 factors are present:  1) Text type is in uppercase and lowercase; 2) Type size is at least 12 point (This is 12 point type); 3) Typographic cues (bold type, color, size of type); 4) No ALL CAPS for long headlines and running text |
| Layout | | *Superior* | At least 5 of the following 8 factors are present:  1) Illustrations are adjacent to the related text; 2) Layout and sequence of information are consistent, making it easy to predict the flow of information; 3) Visual cueing devices (boxes, arrows, shading) are used to direct attention to key content; 4) Pages do not appear cluttered; 5) Use of color supports and is not distracting to the message. Readers need not learn color codes to understand and use the message; 6) Line length is 30 to 50 characters and spaces; 7) There is high contrast between type and paper; 8) Paper has a non-gloss or low-gloss surface |
| Subheadings and “chunking” | | *Not Suitable* | More than 7 items are presented without a subheading (summary page of the BEFORE decision aid) |
| **Learning Stimulation & Motivation** | | | |
| Interaction included in text and /or graphics | | *Superior* | Problems or questions are presented for reader response |
| Desired behavior patterns are modeled | | *NA* |  |
| Motivation | | *Adequate* | Some topics are subdivided to improve readers’ confidence |
| **Cultural Appropriateness** | | | |
| Cultural Match — Logic, Language, Experience (LLE) | | *Superior* | Central concepts of the material appear to be culturally similar to the LLE of the target culture |
| Cultural Image and Examples | | *Superior* | Images and examples present culture in positive ways |
