# Supplementary material for: The "Begin Exploring Fertility Options, Risks and Expectations" (BEFORE) decision aid: development and alpha testing of a fertility tool for premenopausal breast cancer patients
Source: BMC Med Inform Decis Mak. 2019 Oct 28;19:203. doi: 10.1186/s12911-019-0912-y (PMC6819618; doi:10.1186/s12911-019-0912-y)
Supplement: Supplementary file 6 — Additional file 6. International Patient Decision Aid Standards Criteria Assessment of the BEFORE DA. [file 12911_2019_912_MOESM6_ESM.docx]

**Additional File 6.** International Patient Decision Aid Standards Criteria Assessment of the BEFORE (Begin Exploring Fertility Options, Risks, and Expectations) decision aid

| **International Patient Decision Aid Standards Criteria** | | **BEFORE Decision Aid** | |
| --- | --- | --- | --- |
| **Qualifying** | | | |
|  | Q1 Describes health condition or problem for which index decision is required | | 🗸 |
|  | Q2 Explicitly states decision under consideration (index decision) | | 🗸 |
|  | Q3 Describes the options available for the index decision | | 🗸 |
|  | Q4 Describes the positive features of each option | | 🗸 |
|  | Q5 Describes the negative features of each option | | 🗸 |
|  | Q6 Describes the features of options to help patients imagine the physical, social and/or psychological effects | | 🗸 |
| **Certification** | | | |
|  | C1 Shows positive and negative features of options with equal detail Information | | 🗸 |
|  | C2 Provides information about the funding source used for development | | 🗸 |
|  | C3 Provides citations to the evidence selected | | 🗸 |
|  | C4 Provides a production or publication date | | 🗸 |
|  | C5 Provides information about update policy | | 🗸 |
|  | C6 Provides information about the level of uncertainty around outcome probabilities | | 🗸 |
|  | CT1 Describes what the test is designed to measure | | NA |
|  | CT2 Describes next steps taken if test detects a condition/problem | | NA |
|  | CT3 Describes next steps if no condition/problem detected | | NA |
|  | CT4 Describes consequences of detection that would not have caused problems if the screen was not done | | NA |
| **Quality** | | | |
|  | QA1 Development included needs assessment to determine what patients need to make the decision | | 🗸 |
|  | QA2 Development included needs assessment to determine what health professionals need to discuss decision | | 🗸 |
|  | QA3 Development included review by patients not involve in producing the DSI | | 🗸 |
|  | QA4 Development included review by professionals not involve in producing the DSI | | 🗸 |
|  | QA5 DSI was field tested with patients facing the decision | | 🗸 |
|  | QA6 DSI was field tested with practitioners who counsel patients facing the decision | | 🗸 |
|  | QA7 Includes author/developers credentials or qualifications | | 🗸 |
|  | QA8 Evidence that DSI improves match between patient preferences and chosen option | | − |
|  | QA9 Evidence that DSI helps patient improve knowledge about options’ features | | − |
|  | QA10 Describes how research evidence was selected/synthesized | | 🗸 |
|  | QA11 Describes the quality of research evidence used | | 🗸 |
|  | QA12 Provides step by step way to make decision | | 🗸 |
|  | QA13 Includes tools to use when discussing options with practitioner | | 🗸 |
|  | QA14 Describes the natural course of the condition | | 🗸 |
|  | QA15 Makes it possible to compare features of available options | | 🗸 |
|  | QA16 Reports readability levels | | 🗸 |
|  | QA17 Provides information about outcome probabilities (OPs) | | 🗸 |
|  | QA18 Specifies reference class of patient for which OPs apply | | 🗸 |
|  | QA19 Specifies event rates for OPs | | 🗸 |
|  | QA20 Specifies the time period over which OPs apply | | 🗸 |
|  | QA21 Allows to compare OPs using the same denominator | | 🗸 |
|  | QA22 Provides more than one way of viewing probabilities | | 🗸 |
|  | QA23 Asks patients to consider which positive and negative features matter most to them | | 🗸 |
|  | QAT1 Includes information about chances of having a true positive result | | NA |
|  | QAT2 Includes information about chances of having a true negative result | | NA |
|  | QAT3 Includes information about chances of having a false positive result | | NA |
|  | QAT4 Includes information about chances of having a false negative result | | NA |
|  | QAT5 Describes the chance the disease is detected with and without use of the test | | NA |
